# Association of body composition with predicted hip bone strength among Chinese postmenopausal women: a longitudinal study

**DOI:** 10.1038/s41598-019-42031-1

**Published:** 2019-04-02

**Authors:** Xin Shi, Yunyang Deng, Huili Kang, Meng Liu, Yu-Ming Chen, Su-Mei Xiao

**Affiliations:** 1 Department of Medical Statistics and Epidemiology, School of Public Health, Sun Yat-sen University, Guangzhou, 510080 China; 2 Haizhu District Center for Disease Control and Prevention, Guangzhou, 510310 China

## Abstract

Body composition and bone strength are closely associated. How lean mass (LM) and fat mass (FM) contribute to bone strength remains ambiguous. We investigated the associations of total body LM and FM with changes in predicted hip bone strength over a period of 3 years in 1,743 postmenopausal Chinese women from the communities of Guangzhou, China. The body compositions of the women were obtained with dual-energy X-ray absorptiometry. We used the hip structure analysis program to obtain the bone parameters at the femoral neck region, including the bone mineral density (BMD), cross-sectional area (CSA), cortical thickness (CT), section modulus (SM) and buckling ratio (BR). We found the FM and LM were positive predictors for hip bone strength (β > 0, P < 0.05). The LM had a larger contribution to the BMD, CSA, CT, SM and/or their annual percent changes (β_LM_ > β_FM_), while the contribution of FM to the BR and its annual percent change was higher than LM (|β_FM_| > |β_LM_|). Further analysis found that the associations of FM and LM with bone parameters were stronger in the underweight and normal weight participants (|β_BMI1_| > |β_BMI2_|). Overall, FM and LM had positive but differential effects on predicted hip bone strength, with a higher impact in the thinner participants.

## Introduction

Osteoporosis is a common skeletal disorder in middle aged and elderly people^1^. It is characterised by decreased bone strength which can lead to an increased risk of fracture. Bone strength is mainly determined by both bone mass and bone geometric structure. The bone mineral density (BMD) accounts for 50–70% of the total strength of bone^2^. Nonetheless, bone geometry was found to be an independent predictor of fracture risk, and combined with the BMD, the geometry increased the accuracy of a fracture risk prediction model by 13%^3–5^. Studies that examine the BMD and bone geometry together are indispensable for the multidimensional assessment of bone strength and increased accuracy in the prediction of fracture risk.

Body weight has been demonstrated to have a strong relationship with bone strength. Studies have found that underweight individuals had weaker bone strength and a higher fracture risk compared to normal weight individuals^6–8^. Weight gain, such as in overweight or obese individuals, can also have a detrimental effect on bone strength^9,10^. These findings suggest that the relationship between body weight and bone strength might be non-linear^11,12^. The lean mass (LM) and fat mass (FM) together account for approximately 95% of body mass^13^, and these quantities could differentially influence bone strength. The differentiation of the effects of LM and FM could help reveal the underlying mechanisms of body weight on bone strength.

The contribution of LM and FM to bone strength is still ambiguous. Some studies^14–16^ observed an important or primary role of FM in determining bone strength, while some others showed a greater effect of LM on it comparing to FM^17–21^. A favourable effect of LM on bone strength has been identified^22,23^. Several studies have also reported a positive correlation between FM and bone strength^23–25^, but others found no association or even an inverse correlation^26,27^. These inconsistencies could be attributable to population differences in the prevalence of people who are overweight and obese. The association of LM and FM with bone strength varies with obesity status. For example, among 40,050 women and 3,600 men from Canada, the effect of LM on hip bone strength was stronger in non-obese individuals^22^. There are few studies examining body composition and bone geometric structure and even less examining body composition and changes of bone strength.

We investigated the relationships among body composition, quantified with LM and FM, with BMD and the geometric structure of the hip. We also measured how these quantities changed over a period of 3 years in southern Chinese postmenopausal woman. Furthermore, we evaluated the association between body composition and predicted hip bone strength in individuals with varied body mass index (BMI) levels.

## Results

### General participant characteristics

Table 1 shows the basic characteristics for participants at baseline (n = 1,743) and the annual percent changes of the hip bone phenotypes at the 3 year follow-up (n = 1,149). For the women studied at baseline, the median age was 59.3 years and the median years since menopause (YSM) was 9.0 years. There were 75 participants (4.3%) with a BMI below 18.5 kg/m^2^, 981 (56.3%) with a BMI of 18.5–23.9 kg/m^2^, 551 (31.6%) with a BMI of 24.0–27.9 kg/m^2^ and 136 (7.8%) with a BMI over 28.0 kg/m^2^. Approximately 36.7% of the subjects reported taking calcium tablets more than 30 times over the past year. The median values of energy intake and dietary calcium intake were around 1,512.2 kcal/d and 581.5 mg/d, respectively. The mean (standard deviation; SD) values were 20.2 (4.9) kg for FM and 35.5 (4.3) kg for LM. At baseline, the mean (SD) values of the BMD, cross-sectional area (CSA), cortical thickness (CT), section modulus (SM) and buckling ratio (BR) were 0.839 (0.136) g/cm^2^, 2.412 (0.372) cm^2^, 0.162 (0.028) cm, 1.094 (0.212) cm^3^ and 11.000 (2.755), respectively. At the 3 year follow-up, the average annual percent changes for the 1,149 women were −0.506% for the BMD, −0.077% for CSA, −0.561% for CT, −0.050% for SM and −1.619% for BR.

**Table 1:** Basic characteristics of participants (n = 1,743).

Variables	Mean ± SD/Median (25th–75th)/n (%)
Age (years)	59.3 (56.4–63.3)
Height (cm)	154.9 ± 5.3
Weight (kg)	56.2 ± 8.4
**BMI**	
Underweight, <18.5 kg/m^2^ (n, %)	75 (4.3)
Normal weight, 18.5–23.9 kg/m^2^ (n, %)	981 (56.3)
Overweight, 24.0–27.9 kg/m^2^ (n, %)	551 (31.6)
Obesity, ≥28.0 kg/m^2^ (n, %)	136 (7.8)
YSM (years)	9.0 (5.0–13.0)
Physical activity (MET· h/d)^a^	33.1 (30.2–37.1)
Calcium tablets intake (n, %)	640 (36.7%)
Energy intake (kcal/d)	1512.2 (1271.5–1785.7)
Dietary calcium intake (mg/d)	581.5 (442.7–730.5)
**Body composition measures**	
FM (kg)	20.2 ± 4.9
LM (kg)	35.5 ± 4.3
**Bone phenotypes**	
**BMD**	
Baseline (g/cm^2^, n = 1,743)	0.839 ± 0.136
Relative rate of change (%/year, n = 1,149)	−0.506 ± 1.868
**CSA**	
Baseline (cm^2^, n = 1,743)	2.412 ± 0.372
Relative rate of change (%/year, n = 1,149)	−0.077 ± 1.958
**CT**	
Baseline (cm, n = 1,743)	0.162 ± 0.028
Relative rate of change (%/year, n = 1,149)	−0.561 ± 2.015
**SM**	
Baseline (cm^3^, n = 1,743)	1.094 ± 0.212
Relative rate of change (%/year, n = 1,149)	−0.050 ± 2.813
**BR**	
Baseline (n = 1,743)	11.000 ± 2.755
Relative rate of change (%/year, n = 1,149)^b^	−1.619 ± 3.889

Values were presented as mean ± standard deviation (SD), median and interquartile range (25th-75th), or number (n) and percentage (%). ^a^Physical activity including daily occupational activities, leisure time and household chores, was evaluated using metabolic equivalent hours per day (MET· h/d). ^b^Considering BR was negative correlated with bone strength, the relatively rate of BR change (%/year) was calculated as the additive inverse of BR’s change divided by the baseline BR value and the duration of follow-up year. BMI, body mass index; YSM, years since menopause; FM, fat mass; LM, lean mass; BMD, bone mineral density; CSA, cross sectional area; CT, cortical thickness; SM, section modulus; BR, buckling ratio.

### Baseline associations of BMI and body composition with bone phenotypes

**Figure 1:**
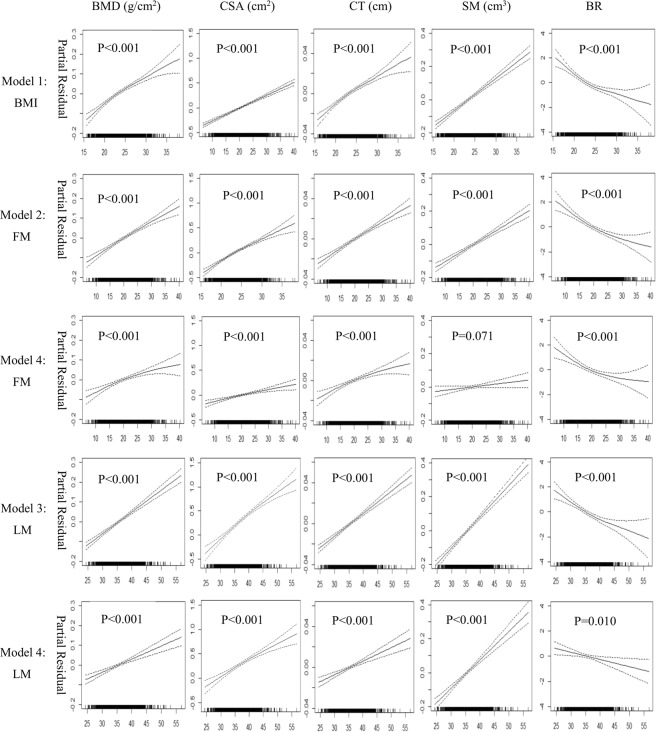
The associations among the BMI, body composition and bone phenotypes at baseline based on the generalised additive regression models (n = 1,743). There were four models: 1. Bone phenotype = f (BMI, covariates); 2. Bone phenotype = f (FM, covariates); 3. Bone phenotype = f (LM, covariates); 4. Bone phenotype = f (FM, LM, covariates). The covariates were age, height, YSM, physical activity, energy adjusted dietary calcium intake and calcium tablets intake. Each graph depicts the association between the independent variable and the dependent variable after removing the influences of the above listed covariates, as well as LM (for Model 4: FM only) and FM (for Model 4: LM only). Dotted lines represented the 95% confidence intervals. BMI, body mass index; YSM, years since menopause; FM, fat mass; LM, lean mass; BMD, bone mineral density; CSA, cross sectional area; CT, cortical thickness; SM, section modulus; BR, buckling ratio.

Figure 1 shows results from the generalised additive regression models (GAMs), which included adjustments for age, height, YSM, physical activity, energy adjusted dietary calcium intake and calcium tablets intake. The BMD, CT and BR exhibited a curvilinear relationship with the BMI (p < 0.001, Model 1). The line became less steep when the BMI was greater than ~25 kg/m^2^. The CSA and SM increased linearly with BMI (p < 0.001, Model 1). The BMD, CSA, CT and SM increased in a near-linear fashion and the BR decreased nonlinearly with FM (p < 0.001, Model 2). After including the LM as a covariate in the model, the relationships of bone variables with the FM became weaker or even disappeared, and the line for the BMD and CT became curved (Model 4). The LM was positively associated with hip bone strength (p < 0.001, Model 3). After the FM was added as a covariate in the model, all the bone phenotypes were linearly related to LM (p < 0.001, Model 4).

The linear regression analyses also identified positive associations of BMI, FM and LM with hip bone strength (p < 0.05, Table 2). The data fits from Model 4 showed LM made a larger contribution to most of the studied bone phenotypes (i.e., BMD, CSA, CT and SM) compared to FM (β_LM_ > β_FM_). The contribution of FM to BR (|β| = 0.153) was larger than LM (|β| = 0.089). The percentage of variation in bone phenotypes explained by the GAMs (R^2^ = 0.130 to 0.400) was only slightly higher than the linear regression models (R^2^ = 0.127 to 0.395).

**Table 2:** Association results of BMI, FM and LM with bone phenotypes at baseline (n = 1,743).

		Linear regression model				GAM Adjusted R^2^
		sβ	SE	P	Adjusted R^2^	
**BMD (g/cm** ^2^ **)**						
Model 1	BMI (kg/m^2^)	0.326	0.001	<**0.001**	0.253	0.256
Model 4	FM (kg)	0.180	0.001	<**0.001**	0.255	0.256
	LM (kg)	0.228	0.001	<**0.001**		
**CSA (cm** ^2^ **)**						
Model 1	BMI (kg/m^2^)	0.385	0.002	<**0.001**	0.387	0.390
Model 4	FM (kg)	0.156	0.002	<**0.001**	0.395	0.400
	LM (kg)	0.329	0.003	<**0.001**		
**CT (cm)**						
Model 1	BMI (kg/m^2^)	0.320	0.001	<**0.001**	0.243	0.246
Model 4	FM (kg)	0.182	0.001	<**0.001**	0.244	0.246
	LM (kg)	0.215	0.001	<**0.001**		
**SM (cm** ^3^ **)**						
Model 1	BMI (kg/m^2^)	0.295	0.001	<**0.001**	0.351	0.353
Model 4	FM (kg)	0.042	0.001	0.102	0.365	0.367
	LM (kg)	0.371	0.001	<**0.001**		
**BR**						
Model 1	BMI (kg/m^2^)	−0.177	0.019	<**0.001**	0.127	0.132
Model 4	FM (kg)	−0.153	0.017	<**0.001**	0.127	0.130
	LM (kg)	−0.089	0.022	**0.010**		

Both in the linear regression model and GAM model, bone phenotype was the dependent variable, and BMI (Model 1), or FM and LM (Model 4) were the predictor variables with age, height, YSM, physical activity, energy adjusted dietary calcium intake and calcium tablets intake as the covariates. P values less than 0.05 were indicated in bold. BMI, body mass index; FM, fat mass; LM, lean mass; BMD, bone mineral density; CSA, cross sectional area; CT, cortical thickness; SM, section modulus; BR, buckling ratio; sβ, standardized β.

The associations of body composition and bone phenotypes in BMI-based subgroups are shown in Table 3. Because the proportions of underweight (4.3%, n = 75) and obese (7.8%, n = 136) of participants were small, the subjects were divided into two subgroups with a BMI of 24.0 kg/m^2^ as the cut-off value. The BMI1 group included the underweight and normal weight individuals. The BMI2 group included the overweight and obese individuals. In both subgroups, the FM was associated with an increased BMD, CSA and CT (β = 0.099 to 0.162, p < 0.05) and decreased BR (β = −0.098 and −0.135, p < 0.05). The LM value was positively related to the BMD, CSA, CT and SM (β = 0.128 to 0.334, p < 0.05). The BR decreased as the LM increased only in the underweight and normal weight group (β = −0.141, p = 0.037). In addition, both the FM and LM contributed more to bone phenotypes in the BMI1 subgroup than the BMI2 subgroup (|β_BMI1_| > |β_BMI2_|).

**Table 3:** Associations between FM, LM and bone phenotypes within each BMI subgroups at baseline (n = 1,743).

	BMI1 (<24.0 kg/m^2^, n = 1,056)			BMI2 (≥24.0 kg/m^2^, n = 687)		
	sβ	SE	P	sβ	SE	P
**BMD (g/cm** ^2^ **)**						
FM (kg)	0.162	0.001	<**0.001**	0.124	0.001	**0.002**
LM (kg)	0.229	0.002	<**0.001**	0.132	0.002	**0.004**
**CSA (cm** ^2^ **)**						
FM (kg)	0.133	0.003	<**0.001**	0.099	0.003	**0.007**
LM (kg)	0.334	0.004	<**0.001**	0.236	0.004	<**0.001**
**CT (cm)**						
FM (kg)	0.162	0.001	<**0.001**	0.126	0.001	**0.001**
LM (kg)	0.221	0.001	<**0.001**	0.128	0.001	**0.006**
**SM (cm** ^3^ **)**						
FM (kg)	0.023	0.002	0.394	0.041	0.002	0.271
LM (kg)	0.331	0.002	<**0.001**	0.235	0.002	<**0.001**
**BR**						
FM (kg)	−0.135	0.028	<**0.001**	−0.098	0.027	**0.018**
LM (kg)	−0.141	0.037	**0.001**	0.003	0.032	0.958

Linear regression analysis was used to detect the associations of FM, LM with bone parameters in each of the BMI subgroups, with the adjustment of age, height, YSM, physical activity, energy adjusted dietary calcium intake and calcium tablet intake. P values less than 0.05 were indicated in bold. FM, fat mass; LM, lean mass; BMD, bone mineral density; CSA, cross-sectional area; CT, cortical thickness; SM, section modulus; BR, buckling ratio; sβ, standardized β.

### Associations of BMI and body composition with the annual percent changes of bone phenotypes at the 3 year follow-up

**Figure 2:**
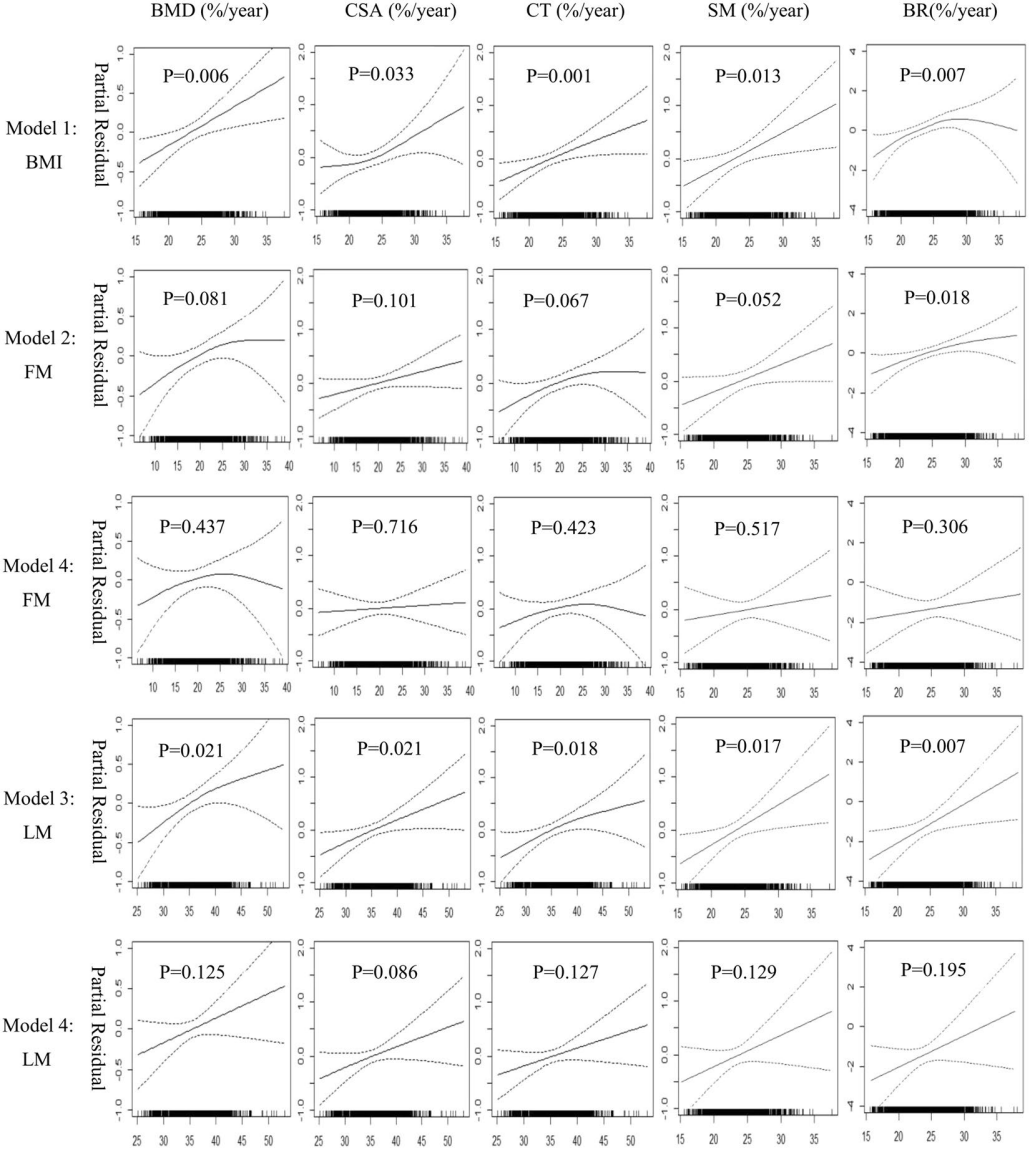
The associations among the BMI, body composition and the annual percent changes of bone phenotypes at the 3 year follow-up based on the generalised additive regression models (n = 1,149). There were four models: 1. Bone phenotype = f (BMI, covariates); 2. Bone phenotype = f (FM, covariates); 3. Bone phenotype = f (LM, covariates); 4. Bone phenotype = f (FM, LM, covariates). The covariates were age, height, YSM, physical activity, energy adjusted dietary calcium intake, calcium tablets intake at baseline and the corresponding baseline value for each bone phenotype. Each graph depicts the association between the independent variable and the dependent variable after removing the influences of the above listed covariates, as well as LM (for Model 4: FM only) and FM (for Model 4: LM only). Dotted lines represented the 95% confidence intervals. BMI, body mass index; YSM, years since menopause; FM, fat mass; LM, lean mass; BMD, bone mineral density; CSA, cross sectional area; CT, cortical thickness; SM, section modulus; BR, buckling ratio.

Figure 2 shows the relationships of BMI, LM and FM with the annual percent changes of bone phenotypes from the GAM analysis, after adjustment for age, height, YSM, physical activity, energy adjusted dietary calcium intake and calcium tablets intake at baseline, as well as the corresponding baseline value for each bone phenotype. The BMI values were almost linearly associated with the increased value of annual percent change of the BMD, CT and SM (p < 0.05, Model 1). The annual percent change of the CSA showed a positive and curved association with BMI (p = 0.033, Model 1). An inverted U-shaped relationship was observed between the BMI and the annual percent change of BR (p = 0.007, Model 1). When the BMI was more than ~25 kg/m^2^, it was negatively associated with the annual percent change of BR. The FM had the only significant relationship with the annual percent change of BR (p = 0.018, Model 2), which was a slightly curved line. The relationship of LM and the annual percent change of BMD and CT was curved, and the LM was linearly related to the annual percent change of CSA, SM and BR (p < 0.05, Model 3). In Model 4, the line trends were similar to the baseline results (Fig. 1), but the associations of FM, LM and the annual percent changes of bone phenotypes were all not significant (p > 0.05).

Similar to the results from the GAMs, the BMI values were significantly associated with the annual percent changes of bone phenotypes at the 3 year follow-up (β = 0.075 to 0.091, p < 0.05; Table 4). In Model 4, which included both the FM and LM, only the LM was related to the annual percent change of CSA and SM (p < 0.05, β = 0.088 and 0.124). The FM was related to the annual percent change of BR (p = 0.002, β = 0.089). No significant associations were observed among the FM, LM and the annual percent changes of bone phenotypes within the two BMI subgroups (p > 0.05, data not shown).

**Table 4:** Association results of BMI, FM and LM with the annual percent changes of bone phenotypes during the 3 years follow-up (n = 1,149).

		Linear regression model				GAM Adjusted R^2^
		sβ	SE	P	Adjusted R^2^	
**BMD (%/year)**						
Model 1	BMI (kg/m^2^)	0.079	0.018	**0.010**	0.052	0.076
Model 4	FM (kg)	0.059	0.012	0.055	0.052	0.076
	LM (kg)	0.019	0.016	0.557		
**CSA (%/year)**						
Model 1	BMI (kg/m^2^)	0.075	0.020	**0.022**	0.037	0.043
Model 4	FM (kg)	0.014	0.016	0.553	0.036	0.041
	LM (kg)	0.088	0.018	**0.018**		
**CT (%/year)**						
Model 1	BMI (kg/m^2^)	0.078	0.020	**0.011**	0.054	0.080
Model 4	FM (kg)	0.057	0.013	0.059	0.054	0.080
	LM (kg)	0.016	0.022	0.559		
**SM (%/year)**						
Model 1	BMI (kg/m^2^)	0.078	0.028	**0.013**	0.046	0.075
Model 4	FM (kg)	0.028	0.023	0.575	0.065	0.074
	LM (kg)	0.124	0.030	**0.001**		
**BR (%/year)**						
Model 1	BMI (kg/m^2^)	0.091	0.036	**0.002**	0.103	0.136
Model 4	FM (kg)	0.089	0.023	**0.002**	0.104	0.133
	LM (kg)	0.036	0.040	0.440		

Both in the linear regression model and GAM model, the annual percent changes of bone phenotype was dependent variable, and BMI (Model 1), or FM and LM (Model 4) were the predictor variables with age, height, YSM, physical activity, energy adjusted dietary calcium intake and calcium tablets intake at baseline, and the corresponding baseline value for each bone phenotype as the covariates. P values less than 0.05 were indicated in bold. BMI, body mass index; FM, fat mass; LM, lean mass; BMD, bone mineral density; CSA, cross sectional area; CT, cortical thickness; SM, section modulus; BR, buckling ratio; sβ, standardized β.

## Discussion

We evaluated the associations of BMI, LM and FM with hip bone phenotypes and their annual percent changes at a 3 year follow-up of 1,743 Chinese postmenopausal women aged 48–77 years. The BMI, LM and FM were all positively associated with predicted hip bone strength. The LM had a larger contribution to the BMD, CSA, CT and SM and/or their annual percent changes. The effect of FM on the BR and its annual percent change was larger than LM. Compared to the BMI1 subgroup, the association strength of LM and FM with bone phenotypes was weaker in the BMI2 subgroup, which included the overweight and obese individuals.

We found a positive relationship between body composition and predicted hip bone strength. The LM was linearly related to bone phenotypes, while the FM had a curved relationship, with the line becoming less steep as FM increased. These results agree with a study of 40,050 Canadian women aged over 50 years. This study found the LM was associated with near-linear increases in hip BMD and CSA, and for the same bone parameters, the FM showed a small initial increase followed by a plateau^22^. There are several possible mechanisms that might explain the different relationships among body composition and bone phenotypes. The LM might benefit bone via muscular contractions and gravitational loading and the favourable endocrine function of skeletal muscles on bone^28^. For example, the insulin-like growth factor 1 (IGF-1) secreted from myofibers could stimulate muscle growth and bone formation through specific receptors or IGF signalling pathways^29^. The impact of FM on bone strength is likely more complicated. FM might have a direct and positive effect on bone through the weight-bearing pathways, while excess adiposity could increase inflammation that leads to bone loss^30^. Adipocytes produce various inflammatory cytokines such as interleukin 6 and tumour necrosis factor alpha. These cytokines might stimulate bone resorption and suppress bone formation through the up-regulation of the receptor activator nuclear factor k ligands^31^. Moreover, the fat-derived hormones could also play important roles in the bone-fat connection, which could bidirectionally influence bone remodelling^32^.

The positive associations among body composition and hip bone phenotypes were weaker in overweight and obese individuals. This finding is broadly consistent with the study by Zhu *et al*. that included 1,014 Caucasian females aged 45–66 years. They found the LM was related to a larger BMD in each tertile of BMI, with regression coefficients lower in the third tertile. In their study, the FM was positively associated with the BMD in the first tertile (BMI ≤ 24.9 kg/m^2^), while the association was weaker or absent in the second (BMI = 25.0–29.3 kg/m^2^) and third BMI tertiles (BMI ≥ 30 kg/m^2^)^33^. In another study, Leslie *et al*.^22^ found the relationships of LM or FM with hip BMD and CSA were stronger in non-obese (BMI < 30 kg/m^2^) than in obese (BMI > 30 kg/m^2^). A possible explanation is that the adaptability of bone on increased weight might decrease with fat gain. Previous studies have reported that being underweight is a risk factor for low bone mass and increased risk of fracture^6,7^. For low-weight and normal-weight individuals, fat could favourably affect bone metabolism, while in overweight and obese individuals, it may become detrimental to bone^34^. During weight loss, studies suggest that exercise should be an important component of weight loss programs to offset the adverse effects of caloric restriction on bone^35^. The different critical points of BMI on bone health identified in previous studies can be attributed to the population differences in the prevalence of overweight and obese individuals.

We found lower LM and FM were independent risk factors for the changes of bone geometric indices of the hip. There are few studies about the relationships among body composition and the changes of bone phenotypes. To the best of our knowledge, this study is the first longitudinal study to specifically examine the associations among body composition and the annual percent change of hip bone phenotypes in Chinese postmenopausal women. There are several studies looking at the relationship among body weight and longitudinal changes in bone geometric indices that support our findings. A 10-year follow-up study of 893 Japanese women aged 18–79 years concluded that an increasing trend in weight was associated with a smaller decline in bone geometric parameters (i.e., CSA and SM) at the hip region^36^. Beck *et al*. investigated the effect of weight change and frailty on hip geometric indices in 4,187 non-black women aged 65 years and older and also found similar results^37^.

An interesting finding in our study is that the FM contributed more to the baseline value and change in hip BR than LM, which differed from the other bone geometric indices we studied. The BR is regarded as an estimate of the cortical stability during buckling. The CSA and CT represent the hip axial compression strength, and the SM is the ability of the hip to bear lateral pressure. Some studies have found that the BR was the best predictor of hip fracture risk compared to the other hip structural parameters, and the BR-predicted fracture risk was equal to BMD^38^. A study with 109 postmenopausal women also found that FM was a stronger determinant of BR in the hip region than LM^39^. Beck *et al*. found that heavier women had lower hip BR values and thus their femurs at the hip were less likely to be compromised by local buckling^37^. These data suggest that FM could be a strong predictor of cortical stability in buckling, and LM could be a strong predictor of hip axial compression strength and bending strength.

The present study was based on a community population, and the findings might generalise well to ordinary urban postmenopausal women in southern China. There are also some limitations to the study. The accurate description of the three-dimensional geometric features of bone is restricted by the inherent limitations of the dual-energy X-ray absorptiometry (DXA) technology. Nevertheless, many studies have found a high correlation among the geometric features described by using the two-dimensional data derived from DXA and a true three-dimensional method^40–42^. Another limitation is the 3 year follow-up might be not long enough to estimate the long-term effects of body composition on changes in bone. Further studies along these lines will be conducted in this ongoing longitudinal study for osteoporosis.

We found both the LM and FM predicted hip bone strength in Chinese postmenopausal women. The LM had a larger contribution to most of the bone variables and/or their annual percent changes. The FM contributed more to the BR and its annual percent change than LM. The protective effects of LM and FM were stronger in thinner individuals. These findings enhance our understanding of the impacts of LM and FM on bone, and imply that the maintenance of a healthy body weight could help to promote the bone strength in Chinese postmenopausal women.

## Materials and Methods

The subjects and data were from the Nutrition and Health Study in Guangzhou, an ongoing cohort study designed to investigate osteoporosis and cardiometabolic outcomes of middle aged and elderly people starting in 2008^8,25,43^. We used data from examinations conducted between June 2010 and July 2017. All participants were residents of urban Guangzhou, China, for more than 5 years. They were recruited by sending invitation letters to residential buildings, posting local advertisements, giving health talks or from referrals in the local community. Individuals were excluded if they had diseases or conditions that might potentially affect bone and mineral metabolism. Exclusion factors included bilateral oophorectomy, parathyroid gland diseases, chronic renal disease, gastrointestinal disease, other skeletal diseases such as rheumatoid arthritis, chronic use of drugs affecting bone metabolism (i.e., hormone replacement therapy, anti-osteoporotic medications) and women with a menopause age before 40 years. A total of 1,743 postmenopausal women aged 48–77 years with baseline bone phenotype measurements of hip and whole body were included in this study. After 3 years (average follow-up period was 3.09 years), there were 1,339 (76.8%) individuals who completed the follow-up survey and phenotype measurement. Among them, 190 individuals were further excluded for having diseases or conditions that might potentially affect bone and mineral metabolism. In total, 1,149 women were included in the longitudinal analysis. This study was approved by the Ethics Committee of the School of Public Health of the Sun Yat-sen University, and written informed consent was obtained from each participant. All research was performed in accordance with relevant guidelines and regulations.

The volunteers were invited to the School of Public Health of Sun Yat-sen University to complete a face-to-face interview^8,25^. The interview was conducted by trained staff using a structured questionnaire that assessed socio-demographic characteristics, disease and medication history, reproductive history and lifestyle habits. The age at menopause was defined as age at the last menstrual period prior to stopping menstruation for twelve months. The YSM was calculated by subtracting the current age from the age at menopause. The daily physical activity (metabolic equivalent, MET) was estimated by asking questions about the frequency and duration of 19 types of activities including sitting and lying^44^. The intake of calcium tablets was defined as taking calcium supplements more than 30 times over the past year. Participant’s height and weight were measured to the nearest 0.1 cm and 0.1 kg, respectively. Weight was measured without shoes and in light clothing. The BMI was calculated as the weight (kg) divided by the height squared (m^2^).

The body composition measures (i.e., total body LM and total body FM) and left hip bone phenotypes were obtained using the DXA (Discovery W; Hologic Inc, Waltham, USA) by the same well-trained professionals. All DXA images of the femoral neck region in hip were used to generate the values of bone phenotypes (i.e., BMD and bone geometry parameters). The bone geometry parameters were calculated by the hip structure analysis (HSA) program included in the APEX software (v3.2, Hologic Inc, Waltham, USA). This method has been described in detail elsewhere^8,25,45^. The bone geometry parameters CSA (cm^2^), CT (cm), SM (cm^3^) and BR were analysed. The SM is an index of resistance to bending forces. The BR describes stable configurations of thin-walled tubes subjected to compressive loads and requires an estimate of the cortical thickness. The relative rates of bone phenotype change (%/year) were calculated as the change of the bone phenotype value divided by the baseline value and the duration (years) of the follow-up. Because the BR was negatively correlated with bone strength, the relative rates of BR change (%/year) were calculated as the additive inverse of BR’s change divided by the baseline BR value and the duration (years) of the follow-up. The precisions of the total body FM and total body LM values were 1.31% and 1.20%, respectively. At the femoral neck region, the *in-vivo* coefficients of variation (CV, %) of BMD, CSA, CT, SM and BR were 1.92%, 1.55%, 2.19%, 2.99% and 4.62%, respectively.

The basic characteristics of the baseline and follow-up subjects were expressed as the mean value and SDs or medians (interquartile range) for the continuous variables and number (percentage) for the categorical variables. The GAMs were used to explore the functional forms of the associations among the BMI, body composition and bone phenotypes. The specific estimation and inference of the relationships were conducted using linear regression models. Four models were used: Model 1. Bone phenotype = f (BMI, covariates); Model 2. Bone phenotype = f (FM, covariates); Model 3. Bone phenotype = f (LM, covariates); and Model 4. Bone phenotype = f (FM, LM, covariates). The linear regression models were also used to analyze the associations of FM, LM with bone parameters in each of the BMI subgroups. The subgroups were defined according to the Working Group on Obesity in China (WGOC)^46^: underweight <18.5 kg/m^2^, normal weight 18.5–23.9 kg/m^2^, overweight 24.0–27.9 kg/m^2^ and obese ≥28.0 kg/m^2^. The confounding factors such as age, height, YSM, physical activity, energy adjusted dietary calcium intake and calcium tablets intake were adjusted in all of the association analyses. The corresponding baseline value of bone phenotype was also added as a covariate in the regression model for the analysis of the 3-year follow-up data. SPSS (v17.0, Chicago, Illinois) and R software (version 3.10, Vienna, Australia) were used for the statistical analyses. A two-sided p-value of less than 0.05 was considered as statistically significant.

## Data Availability

The datasets generated during the current study are not publicly available due to the confidential nature of the material but are available from the corresponding author on reasonable request.

## References

[1] Tarakida A (2011). Hypercholesterolemia accelerates bone loss in postmenopausal women. Climacteric.

[2] Kanis JA (2002). Diagnosis of osteoporosis and assessment of fracture risk. Lancet.

[3] LaCroix AZ (2010). Hip structural geometry and incidence of hip fracture in postmenopausal women: what does it add to conventional bone mineral density?. Osteoporos. Int..

[4] Kaptoge S (2008). Prediction of incident hip fracture risk by femur geometry variables measured by hip structural analysis in the study of osteoporotic fractures. J. Bone Miner. Res..

[5] Faulkner KG (2006). Femur strength index predicts hip fracture independent of bone density and hip axis length. Osteoporos. Int..

[6] Johansson H (2014). A meta-analysis of the association of fracture risk and body mass index in women. J. Bone Miner. Res..

[7] De Laet C (2005). Body mass index as a predictor of fracture risk: A meta-analysis. Osteoporos. Int..

[8] Kang H (2016). Associations of Age, BMI, and Years of Menstruation with Proximal Femur Strength in Chinese Postmenopausal Women: A Cross-Sectional Study. Int. J. Env. Res. Pub. He..

[9] Farr JN (2014). Body composition during childhood and adolescence: relations to bone strength and microstructure. J. Clin. Endocrinol. Metab..

[10] Petit MA (2008). Proximal femur mechanical adaptation to weight gain in late adolescence: a six-year longitudinal study. J. Bone Miner. Res..

[11] Skrzek A, Kozie S, Ignasiak Z (2014). The optimal value of BMI for the lowest risk of osteoporosis in postmenopausal women aged 40–88 years. Homo..

[12] Oldroyd A, Mitchell K, Bukhari M (2014). The prevalence of osteoporosis in an older population with very high body mass index: evidence for an association. Int. J. Clin. Pract..

[13] Hsu YHVSTH (2006). Relation of body composition, fat mass, and serum lipids to osteoporotic fractures and bone mineral density in Chinese men and women. Am. J. Clin. Nutr..

[14] Mizuma N (2006). Difference in the relative contribution of lean and fat mass components to bone mineral density with generation. J. Obstet. Gynaecol. Res..

[15] Lim S (2004). Body composition changes with age have gender-specific impacts on bone mineral density. Bone.

[16] Ijuin M (2002). Difference in the effects of body composition on bone mineral density between pre- and postmenopausal women. Maturitas.

[17] Kim JH (2012). Fat mass is negatively associated with bone mineral content in Koreans. Osteoporos. Int..

[18] Van Langendonck L (2002). Association between bone mineral density (DXA), body structure, and body composition in middle-aged men. Am. J. Hum. Biol..

[19] Taaffe DR (2001). Race and sex effects on the association between muscle strength, soft tissue, and bone mineral density in healthy elders: the Health, Aging, and Body Composition Study. J. Bone Miner. Res..

[20] Khosla S (1996). Relationship between body composition and bone mass in women. J. Bone Miner. Res..

[21] Reid IR, Plank LD, Evans MC (1992). Fat mass is an important determinant of whole body bone density in premenopausal women but not in men. J. Clin. Endocrinol. Metab..

[22] Leslie WD (2014). Estimated lean mass and fat mass differentially affect femoral bone density and strength index but are not FRAX independent risk factors for fracture. J. Bone Miner. Res..

[23] Hu WW (2012). Lean mass predicts hip geometry and bone mineral density in chinese men and women and age comparisons of body composition. J. Clin. Densitom..

[24] Hong X (2010). Percent fat mass is inversely associated with bone mass and hip geometry in rural Chinese adolescents. J. Bone Miner. Res..

[25] Han GY (2017). Fat Mass Is Positively Associated with Estimated Hip Bone Strength among Chinese Men Aged 50 Years and above with Low Levels of Lean Mass. Int. J. Env. Res. Pub. He..

[26] Kang DH (2015). Association of body composition with bone mineral density in northern Chinese men by different criteria for obesity. J Endocrinol. Invest..

[27] Zhao LJ (2007). Relationship of obesity with osteoporosis. J. Clin. Endocrinol. Metab..

[28] Lanyon LE, Rubin CT (1984). Static vs dynamic loads as an influence on bone remodelling. J. Biomech..

[29] Hamrick MW (2010). Role of muscle-derived growth factors in bone formation. J. Musculoskelet. Neuronal. Interact..

[30] Braun T, Schett G (2012). Pathways for bone loss in inflammatory disease. Curr. Osteoporos. Rep..

[31] Pal, C. S., Sanyal, S. & Chattopadhyay, N. Adiponectin signaling and its role in bone metabolism. *Cytokine* (2018). 10.1016/j.cyto.2018.06.012 29937410

[32] Elefteriou F (2005). Leptin regulation of bone resorption by the sympathetic nervous system and CART. Nature.

[33] Zhu K (2015). Associations between body mass index, lean and fat body mass and bone mineral density in middle-aged Australians: The Busselton Healthy Ageing Study. Bone.

[34] Ilich JZ (2014). Interrelationship among muscle, fat, and bone: connecting the dots on cellular, hormonal, and whole body levels. Ageing. Res. Rev..

[35] Villareal DT (2006). Bone mineral density response to caloric restriction-induced weight loss or exercise-induced weight loss: a randomized controlled trial. Arch. Intern. Med..

[36] DongMei N (2012). Association between weight changes and changes in hip geometric indices in the Japanese female population during 10-year follow-up: Japanese Population-based Osteoporosis (JPOS) Cohort Study. Osteoporos. Int..

[37] Beck TJ (2001). Structural adaptation to changing skeletal load in the progression toward hip fragility: the study of osteoporotic fractures. J. Bone Miner. Res..

[38] Gnudi S, Sitta E, Fiumi N (2007). Bone density and geometry in assessing hip fracture risk in post-menopausal women. Br. J. Radiol..

[39] El HR, Baddoura R (2012). Anthropometric predictors of geometric indices of hip bone strength in a group of Lebanese postmenopausal women. J. Clin. Densitom..

[40] Khoo BC (2012). Differences in structural geometrical outcomes at the neck of the proximal femur using two-dimensional DXA-derived projection (APEX) and three-dimensional QCT-derived (BIT QCT) techniques. Osteoporos. Int..

[41] Ramamurthi K (2012). An *in vivo* comparison of hip structure analysis (HSA) with measurements obtained by QCT. Osteoporos. Int..

[42] Khoo BC (2009). Comparison of QCT-derived and DXA-derived areal bone mineral density and T scores. Osteoporos. Int..

[43] Cao Y (2016). Association of magnesium in serum and urine with carotid intima-media thickness and serum lipids in middle-aged and elderly Chinese: a community-based cross-sectional study. Eur. J. Nutr..

[44] Wang P (2012). Association of natural intake of dietary plant sterols with carotid intima-media thickness and blood lipids in Chinese adults: a cross-section study. PLoS One.

[45] Yang L (2009). Use of DXA-based structural engineering models of the proximal femur to discriminate hip fracture. J. Bone Miner. Res..

[46] Qi XQ (2004). The Guidelines for Prevention and Control of Overweight and Obesity in Chinese Adults - Foreword. Biomed. Eeviron. Sci..

